# Hair Growth Promoting Activity of Cedrol Nanoemulsion in C57BL/6 Mice and Its Bioavailability

**DOI:** 10.3390/molecules26061795

**Published:** 2021-03-23

**Authors:** Yaling Deng, Feixue Huang, Jiewen Wang, Yumeng Zhang, Yan Zhang, Guangyue Su, Yuqing Zhao

**Affiliations:** 1 Traditional Chinese Medicine College, Shenyang Pharmaceutical University, Shenyang 110016, China; yl18341473234@163.com (Y.D.); huangfeixue1@163.com (F.H.); 18149393318jw1@163.com (J.W.); zym122908@126.com (Y.Z.); lx1601747244@163.com (Y.Z.); 2 Key Laboratory of Structure-Based Drug Design and Discovery of Ministry of Education, Shenyang Pharmaceutical University, Shenyang 110016, China

**Keywords:** cedrol, nanoemulsion, hair growth, pharmacokinetics, pharmacodynamics

## Abstract

As the main component of *Platycladus orientalis*, cedrol has known germinal activity. A range of cedrol formulations have been developed to prevent hair-loss, but compliance remains key issues. In this study, we prepared cedrol nanoemulsion (CE-NE) and determined the particle size and PDI (polydispersion coefficient), investigated the hair growth activity and studied the bioavailability in vitro and in vivo. Results showed that the average particle size of CE-NE is 14.26 ± 0.16 nm, and the PDI value is 0.086 ± 0.019. In vitro drug release investigation and drug release kinetics analysis showed release profile of CE from nanoparticles demonstrates the preferred partition of CE in buffer pH 4.0, the release profile of CE-NE showed a first-order kinetics reaching around 36.7% after 6 h at 37 °C. We artificially depilated the back hair of C57BL/6 mice and compared the efficacy of a designed cedrol nanoemulsion to an existing ointment group. The hair follicles were imaged and quantified using a digital photomicrograph. The results showed that compared with the ointment, CE-NE had positive effects on hair growth, improved drug solubility. Compared with the ointment and 2% minoxidil groups, 50 mg/mL CE-NE led to more robust hair growth. Pharmacokinetics analysis showed that the AUC_0–*t*_ of CE-NE was 4-fold higher than that of the ointment group, confirming that the bioavailability of the nanoemulsion was greater than that of the ointment. CE-NE also significantly reduced the hair growth time of model mice and significantly increased the growth rate of hair follicles. In conclusion, these data suggest that the nanoemulsion significantly improved the pharmacokinetic properties and hair growth effects cedrol, enhancing its efficacy in vitro and in vivo.

## 1. Introduction

Alopecia is a common skin disease, which significantly affects mental health and quality of life. Amongst its disease subtypes, congenital alopecia, alopecia and androgenic alopecia are most common [1]. An increasing number of alopecia cases are emerging due to a range of factors, including impaired immune function, skin diseases, and environmental factors [2,3]. The current anti-alopecia drugs used in the clinic are finasteride and minoxidil which despite their therapeutic effects, producing unpredictable side effects [4,5], including dizziness, cardiovascular disease and allergic dermatitis. New treatments that lack these toxicity issues remain a major focus of Chinese and Western medicine [1]. In recent years, natural products have emerged as effective sources of novel anti-alopecia agents [6].

*Platycladus orientalis* is an evergreen tree in the cupressaceae family that according to pharmacopoeia, the leaves and seeds of which holds medicinal value [7]. Cedrol (CE), one of the active ingredients in *Platycladus orientalis*, is a high-boiling-point sesquiterpineol, which show anti-tumor, anti-inflammatory, analgesic and regulating cardiovascular effects [8,9,10,11,12]. Moreover, cedrol, this natural product has also been reported as a hair follicle platelet activating factor antagonist [13,14], that can increase the blood flow around the hair follicles, and enhance the transport of nutrients and oxygen to hair follicle cells [15]. Nanoemulsions are submicron colloidal dispersion systems with a mean particle size of 10 and 100 nm. Nanoemulsions have recently been used to overcome the formulation issues of poorly soluble drugs and have attracted wide attention from the pharmaceutical industry. Cedrol is one such example that has been used as a nanoemulsion preparation through the addition of excipients. Due to their small particle size, fast transdermal absorption, and rapid penetration into the skin, nanomaterials have drug delivery advantages over conventional drug dosage regimens [16].

Preliminary studies in our laboratory found that the ointment formulation of cedrol has a hair growth promotion effect, gave no irritation and were safe for topical administration. However, when the ointment is applied to the affected area, it is easy to pollute the clothing, which greatly reduced the patient’s compliance. In addition, the efficacy is limited by the transdermal ability of the preparation [17,18]. In this study, we investigated the efficacy of cedrol ointment, cedrol nanoemulsions (CE-NE) (25 mg/kg, 50 mg/kg and 100 mg/kg) to identify the most efficient drug preparation. C57BL/6 mice in the telogen phase were selected as an animal model of hair growth, due to their characterized and dorsal hair time-synchronous growth cycle. We investigated the effects of cedrol on hair growth in vivo, through the investigation of hair length, hair weight and hair follicle morphology [19]. In view of the existing problems, this study made use of the characteristics and advantages of nanoemulsion to prepare nanoemulsion transdermal delivery form. It is expected that the prepared cedrol nanoemulsion can improve the efficacy of cedrol and have a good transdermal absorption effect. Reduce the dosage, so as to make the clinical application more convenient and effective.

## 2. Results

### 2.1. Evaluation of CE-NE Type, Emulsion Size, and Zeta Potential

**Figure 1:**
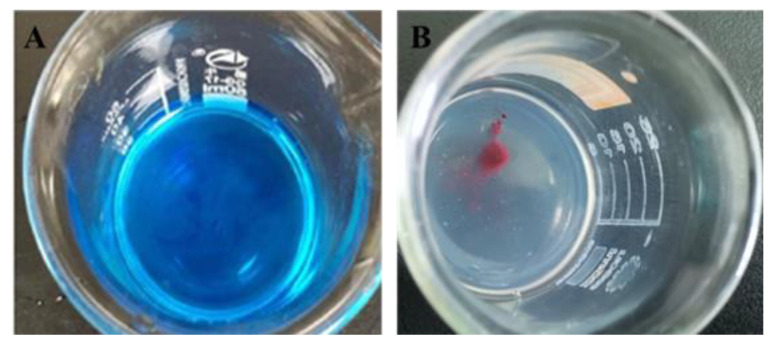
Evaluation of CE-NE type, (**A**) is diffusion after the addition of methylene blue, (**B**) is diffusion after the addition of Sudan red.

Figure 1A,B show the diffusion of the dye solution in the nanoemulsion system after the addition of a drop of methylene blue and Sudan red, respectively. The diffusion rate of blue dye is obviously faster than that of red dye, which indicates that the nanoemulsion is O/W type.

**Figure 2:**
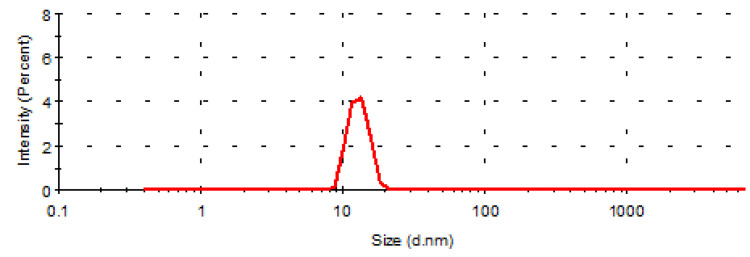
Evaluation of emulsion size.

The particle size and polydispersion coefficient (PDI) of nanoemulsions are important indexes to evaluate nanoemulsions. The particle size and PDI of the nanoemulsion measured by Nano ZS90 laser particle size analyzer are shown in Figure 2, the average particle size of CE-NE is 14.26 ± 0.16 nm, and the PDI value is 0.086 ± 0.019. The particle size distribution of nanoemulsion is uniform before and after drug loading.

### 2.2. In Vitro Drug Release Investigation and Drug Release Kinetics Analysis

**Figure 3:**
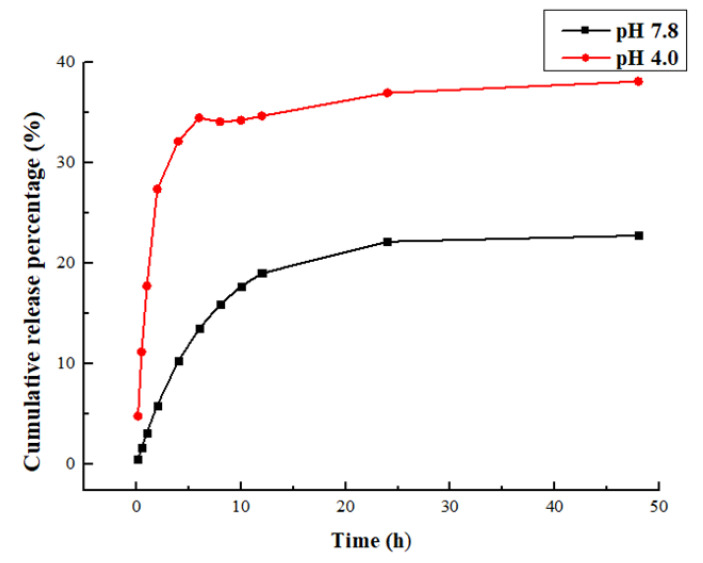
Cumulative release curve.

The release kinetics of CE at the studied conditions is shown in Figure 3. The phosphate buffer (pH 4.0) was used to provide the sink condition. The treatment of CE-NE showed a quick increase to 36.7% in 6 h release, which reflects the diffusion of CE from the dialysis back to the outer sink. This was followed by a much smaller increase rate in the subsequent hours to achieve the highest release of 38.4% (Figure 3). In contrast, the release rate of CE from the phosphate buffer (pH 7.8) was much lower than phosphate buffer (pH 4.0). After 48 h, approximately 22.6% of CE was released from the nanoemulsion, which is lower than the pH 7.8 with 15.8% release. The slow release profile of CE from nanoparticles demonstrates the preferred partition of CE in nanoemulsion with buffer pH 4.0.

The in vitro release equations of CE from nanoemulsion were listed in Table 1 and Table 2. The goodness of fit R^2^ of the zero-order dynamic equation, Higuchi dynamics and Korsmeyer–Peppas dynamics of CE-NE are 0.2701, 0.5621 and 0.7774, respectively, which are all not very good. Additionally, the first-order kinetics equation of goodness-of-fit is 0.9854, close to 1; the data revealed the sustained release profile of CE fitted best to First-order kinetics equation. The release behavior of the encapsulated drug could be affected by the encapsulation pattern and surface properties. The normal scalp environment pH is 4.5–6.5, while the medium oil scalp will show acidic scalp environment (pH 3–4.5); dry scalp (pH 7–9); based on the observed release profile and equation, it could be deduced that the CE release slowly by means of dissolution and diffusion, and CE-NE is more effective for the control of hair loss in the medium oil environment.

### 2.3. Evaluation of Hair Growth Cycle

**Figure 4:**
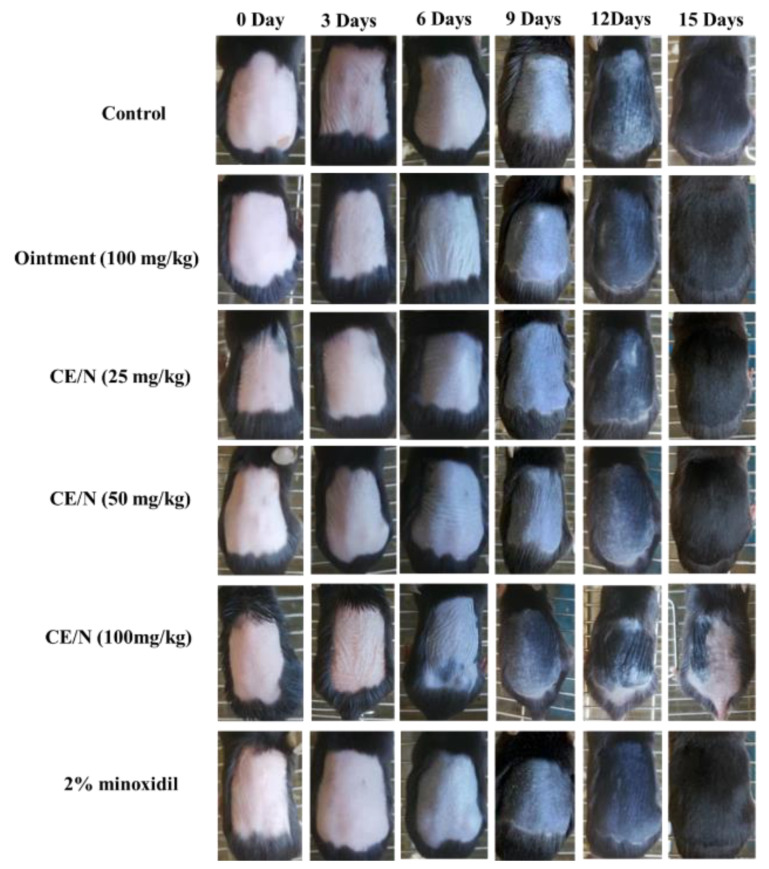
Effects of topical cedrol on hair regeneration in C57BL/6 mice. In vivo hair regeneration was induced by depilating the hairs with a wax/rosin mixture in 6 week-old C57BL/6 mice. The back skin were topically applied with ointment, CE-NE (25 mg/kg, 50 mg/kg and 100 mg/kg), 2% minoxidil (*n* = 10). Photographs were taken on 0, 3, 6, 9, 12, 15 days after applying cedrol on the shaved dorsal skin.

The characteristic hair growth patterns of the model mice are shown in Figure 4. At the beginning of the growth period, melanin deposition in the skin appeared gray. On the basis of the hair growth states of C57BL/6 mice, an interval of 3 days was selected for image acquisition. After 5 days of administration, the skin color of the mice changed from pink to light gray. On day 11, the epidermis of the mice in each group turned dark gray and fine hair appeared. CE-NE (100 mg/kg) mice showed alopecia on day 12 due to the excessive dosing leading to toxic side effects.

**Figure 5:**
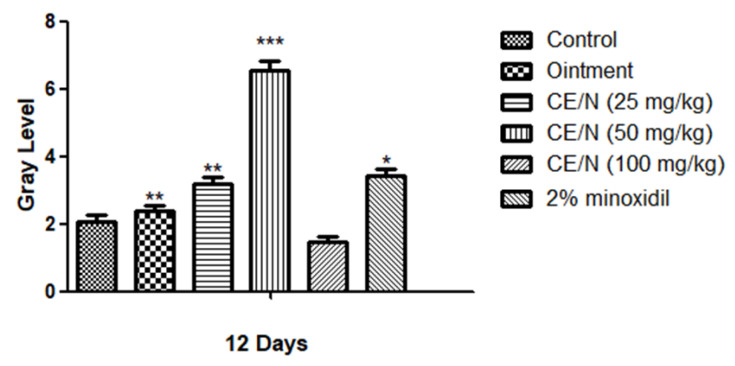
Grayscale analysis in alopecia. The back skin were topically applied with ointment, CE-NE (25 mg/kg, 50 mg/kg and 100 mg/kg), 2% minoxidil. Photographs and analyzes were taken on 12 days after applying cedrol on the shaved dorsal skin. The results were shown as the mean values ± S.D. * *p* < 0.05, ** *p* < 0.01, *** *p* < 0.001, when compared to respective control values by Student’s *t*-test (*n* = 6).

On day 15, the entire depilation area on the back of the mice was covered with new hair and no new hair growth could be observed. Mice in the 100 mg/kg CE-NE group regained fine hair after 19 days. After the cedrol treatment, the gray value of hair was significantly increased in CE/NE groups, and showed a dose-dependent relationship (Figure 5).

The time required for each growth stage is shown in Table 3. After 7–9 days of application, the skin of the mice in the blank group appeared gray compared to 6-8 days post administration in the 2% minoxidil group and 5–6 days post administration in the ointment group. Compared to the minoxidil group (7.5 ± 0.8 day) and ointment group (6.75 ± 0.6 day), the hair growth time of the CE-NE (50 mg/kg) group (6.2 ± 0.4 day) significantly improved. The results showed that CE-NE (50 mg/kg) promoted rapid hair growth during the initial stages. As shown in Table 3, the beneficial effects of the CE-NE 50 mg/kg group (11.8 ± 0.4 day) occurred significantly earlier, suggesting that CE-NE could effectively reduce the time for hair to enter the growth period.

### 2.4. Determination of Hair Length and Weight

**Figure 6:**
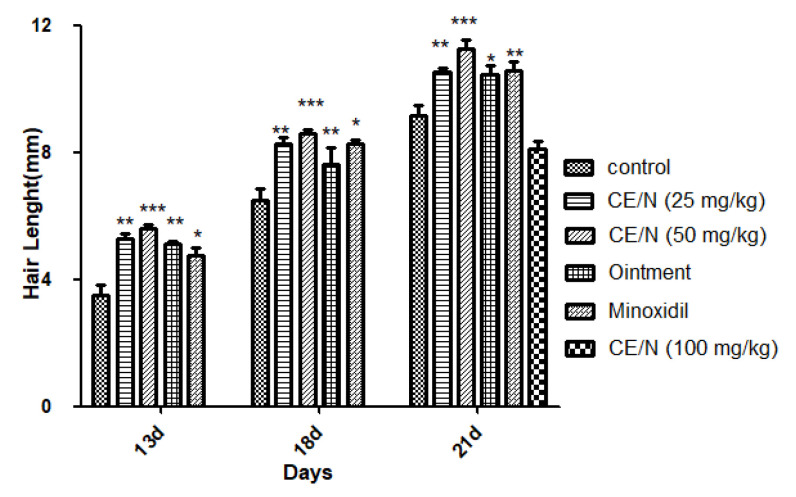
Hair length of mice at different time after beginning the treatment of CE-NE, 2% minoxidil and ointment. Results were showed as mean standard deviation (S.D). (* *p* < 0.05, ** *p* < 0.01, *** *p* < 0.001), compared with the control (*n* = 10).

From 14 days post administration, the entire depilation area on the back of the mice was covered with newly grown hair, and the growth of new hair could no longer be observed by appearance. Therefore, we evaluated hair growth status by measuring the length and weight of new hair. As shown in Table 4 and Figure 6, mice treated with 50 mg/mL CE-NE produced a greater effect on hair length and growth compared to the control and 2% minoxidil groups, particularly on days 16 and 21. The hair length of the 50 mg/mL CE-NE group (11.3 ± 0.7 mm) was also significantly longer than that of the ointment and 2% minoxidil groups on day 21, which were 10.5 ± 0.7 mm and 10.6 ± 0.7 mm, respectively.

We further measured the weight of newly grown hair in all groups (Table 5). Hair weights of the mice treated with 50 mg/mL CE-NE were the heaviest with values of 0.54 ± 0.04 mg. The gross weights of mice in the control, ointment, CE-NE (25 mg/mL and 100 mg/kg) and 2% minoxidil group were 0.22 ± 0.06, 0.44 ± 0.04, 0.41 ± 0.08, 0.46 ± 0.04, and 0.20 ± 0.31 mg, respectively. Compared with the 2% minoxidil and control groups, the weight of the dorsal fur in the 50 mg/mL CE-NE group was heavier.

### 2.5. Histological Observation of Hair Follicles

**Figure 7:**
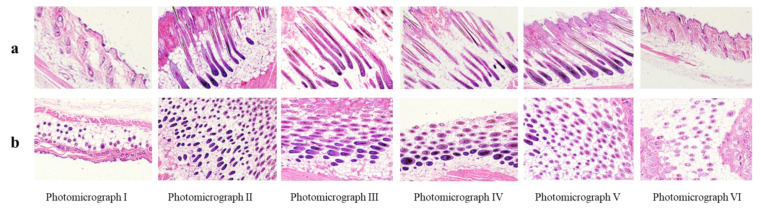
Effects of topical CE-NE, 2% minoxidil and ointment on hair follicles in C57BL/6 mice. Photographs for different groups in mice (**a**) and vertical section (**b**) horizontal section. Photomicrograph I: hair follicles in blank control group; Photomicrograph II: hair follicles in CE-NE (25 mg/mL) group; Photomicrograph III: hair follicles in CE-NE (50 mg/mL) group; Photomicrograph IV: hair follicles in ointment group; Photomicrograph V: hair follicles in 2% minoxidil group; VI: hair follicles in CE-NE (100 mg/ mL) group. (*n* = 6).

Hair follicles were immediately imaged and analyzed following their plucking from the shaved area. The data indicated that CE-NE (50 mg/kg) could stimulate the growth of the hair follicles. As shown Figure 7, the cyclic phase of hair growth was markedly influenced by cedrol and 2% minoxidil treatment. In the control groups, most hair follicles were in the telogen phase, with low numbers in the catagen phase, and nearly no follicles in the anagen phase. However, the length of hair follicles in the CE-NE (25 mg/mL and 100 mg/kg) group and ointment group were less than that in the CE-NE (50 mg/mL) group. In general, in the CE-NE (50 mg/mL) group, most hair follicles were in the anagen phase.

Treatment with 50 mg/mL CE-NE in the mice led to a more remarkable effect on hair follicle length than those of the control, ointment or 2% minoxidil groups (Table 6). The CE-NE groups (25 and 50 mg/mL) attained lengths of 209.99 μm and 236.18 μm, compared to 55.22 μm in the control group.

### 2.6. Pharmacokinetic Evaluation

**Figure 8:**
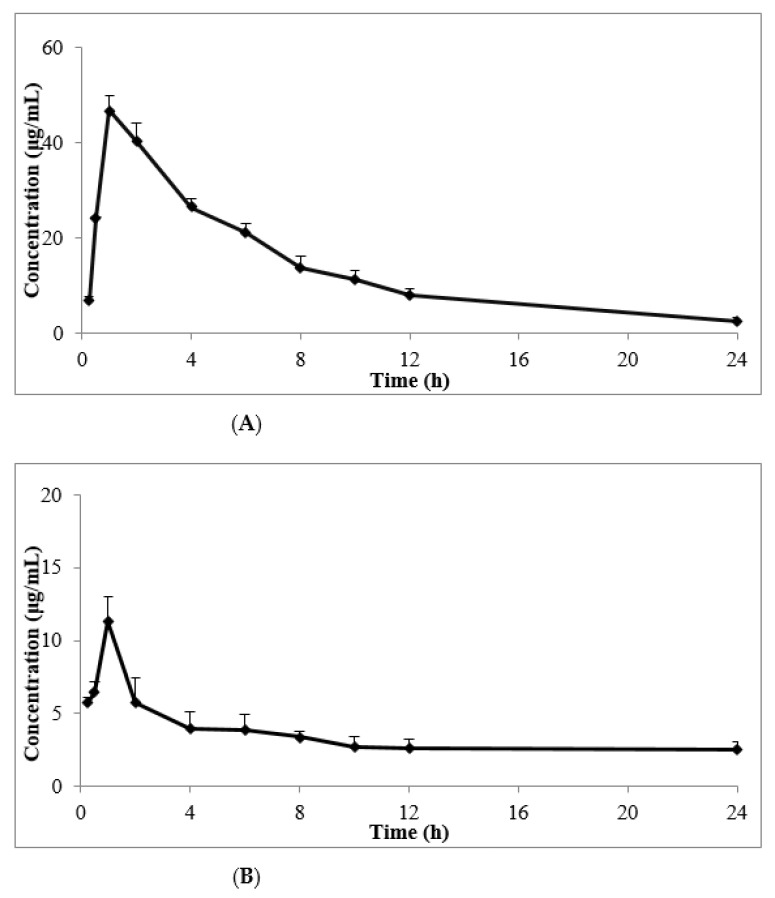
The mean drug–time curves of skin tissues after topical administration of CE-NE (**A**) and ointment (**B**) in KM mice. (*n* = 5).

Drug–time curves showed that following the topical use of 50 mg/kg, an obvious absorption phase was observed, and the skin concentrations of cedrol significantly increased during the initial stage of administration (Figure 8). The effects of both the CE-NE and ointment were rapid, and the peak time of the CE-NE showed no obviously significant differences compared to that of the ointment. As shown in Table 5, the clearance (CL) of the CE-NE (141.44 ± 12.30 L/h) was 2/5-fold lower than that of the ointment (353.35 ± 178.55 L/h). The mean residence time (MRT) (0–*t*) of the CE-NE (9.63 ± 1.04 h) was 1.4-fold longer than that of the ointment (6.72 ± 0.17 h). In addition, the area of the concentration-time curve (AUC) (0–*t*) of the CE-NE (330.30 ± 23.07 μg/mL·h) was 4-fold higher than that of the ointment (82.56 ± 6.52 μg/mL·h). This may be because the nanoemulsion increased the effect of drug adhesion on the biological mucosa and prolonged the retention time, allowing completely drug released at the absorption site, thus improve its bioavailability.

## 3. Discussion

The results of drug release in vitro showed that CE-NE had good drug release characteristics in the buffer of pH4.0 (Figure 3), and the free CE rapidly increased to 36.7% within 6 h, compared with 22.6% in the buffer of pH 7.8. It can be concluded that the acidic environment is for the medium solution to enter the nanoparticles and dissolve the drug.

The data revealed the sustained release profile of CE fitted best to First-order kinetics equation, and the equation of goodness-of-fit is 0.9854, close to 1 (Table 1). The drug is released quickly at first, and then slowly, which may be due to the adsorption of CE, on the surface of the nanoparticles, so the drug is released faster, so that more drugs can be released quickly after reaching the target site, reaching a higher concentration. With the progress of release, the drug encapsulated in the nanoparticles is gradually released because of the dissolution of the carrier material, and the drug release rate is relatively slow to maintain the efficacy, which is beneficial to improve the drug utilization rate.

Hair follicles undergo a cycle of anagen, catagen, and telogen, and re-arrangement of the skin vasculature occurs during hair cycling [20,21,22]. Minoxidil, as a chemical drug, has been used to stimulate hair growth for many years [23]. However, its general use is limited due to the high occurrence of side effects. It is therefore of importance to develop new therapeutic drugs to prevent hair loss and promote hair growth. The cedrol extract of *Platycladus orientalis* shows promising effects on hair growth. In this study, we compared the efficacy of cedrol ointment to it nanoemulsion form in C57BL/6 mouse models. The ointment forms a semi-solid preparation that is sticky, shows poor fluidity, and does not spread evenly. Its ability to penetrate the skin barrier is weak, and the retention time on the surface of the skin is longer. In contrast, the nanoemulsion is thermostable transparent liquid and small particle size and easy absorption after application, which can improve the compliance of patients; and from the drug time curve, the amount of cedrol absorbed is also larger than that of ointment. Therefore, nanoemulsion can protect the drug and improve its transdermal absorption [24,25,26]. Therefore, as a new dosage form, nanoemulsions should be paid more attention for future cedrol dosing regimens.

We evaluated the effects of ointment and CE-NE, on hair growth using various methods. As shown in Figure 4 and Table 3, mid-dose nanoemulsions (50 mg/mL) successfully reduced the time required for hair growth initiation compared with the ointment and 2% minoxidil groups. The hair lengths and weights in all groups were also evaluated (Table 4 and Table 5). The hair lengths in the mid-dose nanoemulsion groups were longer than those of the ointment and 2% minoxidil groups. The hair weight of the CE-NE (50 mg/kg) group was significantly higher than that of the ointment and minoxidil group.

The growth and regeneration of hair is dependent rich blood supply to hair follicles. Disorders of microcirculation perfusion leads to decreased blood perfusion speed and microcirculation blood flow, which influences the normal oxygen supply of scalp hair follicles and leads to alopecia [1]. Vascular endothelial growth factor (VEGF), is a specific heparin binding growth factor of vascular endothelial cells, that can effectively promote vascular regeneration [27]. CE promotes the secretion of vascular endothelial growth factor VEGF in hair follicles [28]. CE also regulates intracellular signaling, and is a known inducer of MAPK protein [29]. We therefore speculate that CE enhances hair growth through its ability to increase the blood flow around the hair follicles and to transfer oxygen and nutrients to hair follicle cells. The effects of both the CE-NE and ointment were rapid, with the peak time of CE-NE showing no significant differences to that of the ointment. As shown in Table 7, the clearance (CL) of the CE-NE (141.44 ± 12.30 L/h/kg) was 2/5-fold lower than that of the ointment (353.35 ± 178.55 L/h/kg).Whilst the mean residence time (MRT) (0–*t*) of the CE-NE (9.63 ± 1.04 h) was 1.4-fold longer than that of the ointment (6.72 ± 0.17 h),In addition, the area of the concentration-time curve (AUC) (0–*t*) of the CE-NE (330.30 ± 23.07 μg/mL·h) was 4-fold higher than that of the ointment (82.56 ± 6.52 μg/mL·h),suggesting that its bioavailability was 4 times of that of ointment group and the absorption was faster.

Taken together, these data highlight that the bioavailability of nanoemulsions of poorly water-soluble compounds is higher than that of ointments improving drug absorption and efficacy. It is likely that these effects are due to improved pharmacokinetics and tissue distribution, characterized by a prolonged residence time in vivo after the topical administration of the nanoemulsion.

## 4. Experimental

### 4.1. Materials

CE-NE and ointment were made in the laboratory, Purity > 98%. A 2% minoxidil solution was purchased from a local market. XO-250 ultrasonic cell breaker (Nanjing Xianou Instrument Manufacturing Co. LTD, Nanjing, China.)

### 4.2. Animals

4-week-old C57BL/6 mice (SPF grade), weighing 20 ± 2 g, female, animals were raised in separate cages. Provided by the Experimental Animal Center of Shenyang Pharmaceutical University. Laboratory Animal Certificate No: SYXK (Liao) 2018-0009. The experimental animals were placed in polypropylene cages (12 h light / dark cycle, 23 ± 2 °C and 35–60% humidity). All animals have access to water and food at will. All the experimental procedures in this study were operated in accordance with the guidelines for the use of experimental animals in Shenyang Pharmaceutical University, and the number of animals was kept to a minimum.

### 4.3. Preparation of CE-NE and Ointment

CE-NE: Weigh the CE powder through a 100-mesh sieve, add the weighed oil phase (including medium chain oil and Span 80), and heat the water bath to 70 °C and keep the temperature. Take the prescription amount of water phase (Tween 80, potassium sorbate, distilled water), heat the water bath to 70 °C, use a magnetic agitator to add the oil phase to the water phase drop by drop, stir 60 min to make colostrum. When colostrum is cooled to room temperature, ice bath probe ultrasonic 12 min (ultrasound 3 s, interval 3 s), natural cooling to room temperature.

Ointment: Weigh CE powder through 100 mesh sieve, add the oil phase (including white Vaseline, stearic acid, glycerol monostearate, liquid paraffin), heat the water bath to 80 °C and keep the temperature. The weighing water phase (glycerin, Tween 80, deionized water) was stirred evenly at 80 °C. The oil phase was added to the water phase drop by drop using a magnetic agitator (400 rpm), and the 30 min was stirred. Take out the beaker, stir it with a glass rod at a uniform speed to make it ointment, and naturally cool to room temperature.

### 4.4. Determination of the Type of Nanoemulsion, Emulsion Size, and Zeta Potential

Add equal amount of Sudan red (oil-soluble dye) and methylene blue (water-soluble dye) to the nanoemulsion shake it gently; observe the diffusion rate in the nanoemulsion to determine the type. If the red dye diffuses faster than the blue, it is O/W type nanoemulsion; otherwise, it is O/W type.

The CE-NE was diluted with distilled water to an appropriate concentration, and then added to the sample pool of Malvin particle size detector for determination. The sample was, respectively repeated three times to obtain the average emulsion size, and zeta potential of the nanoemulsion (mean ± SD).

### 4.5. In Vitro Drug Release Investigation and Drug Release Kinetics Analysis

A certain amount of CE-NE was placed in a pre-treated dialysis bag, both ends of which were clamped together and placed in a beaker. The release media were 100 mL of pH −4.0 and pH −7.8 phosphate buffer, respectively, and stirred slowly at a constant speed at 37 °C water bath temperature, and the rotating speed was 50 rpm/min. At 0.5, 1, 2, 4, 6, 8, 10, 12, 24 and 48 h, 4 mL of release medium was taken, filtered, and the same amount of release medium was added. GC was determined according to the gas phase condition, and the cumulative drug release curve was drawn.

#### GC Conditions

Chromatographic separation was performed by using a 6890 GC-FID gas chromatographic system (Agilent, Palo Alto, Santa Clara, CA, USA). Chromatographic separation was achieved on a HP-5MS column (30 m × 0.32 mm × 0.25 um) purchased from America. Cedrol was analyzed by GC using nitrogen as the carrier gas with a flow of 1.0 mL/min. The initial column temperature was held at 130 °C for 1 min, then increased at a rate of 10 °C min^−1^ to 150 °C for 2 min, and then increased at a rate of 10 °C min^−1^ to 230 °C for 1 min. The split ratio is 10:1.

### 4.6. Grouping and Modeling

#### 4.6.1. Effect of CE /NE on Hair Growth in C57BL/6 Female Mice

Eighty-four female mice with a body weight of 20 ± 2 g were randomly divided into 5 groups, with 12 mice in each group. The patients were treated with blank nanoemulsion, external CE-NE (25 mg/kg, 50 mg/kg and 100 mg/kg), ointment and 2% minoxidil, respectively, and the control group was treated with blank nanoemulsion. For 21 consecutive days, and the medicine was given twice a day (12 h apart).

#### 4.6.2. Establishment of Hair Loss Model of C57BL/6 Female Mice with CE-NE

The mice were acclimation to feeding for 3 days. Intraperitoneal injection of 0.2 mL/20 g of prepared chloral hydrates (about 35min for anesthesia). Rosin and paraffin wax were heated and melted at 1:1 and then mixed and soaked in 2 cm × 2 cm gauze. After cooling to an appropriate temperature, they were removed and covered on the back of the anesthetized mice. After cooling and solidification, the hair was removed, and the mice hair follicles were induced from the quiescent stage to the growing period. On the second day of depilation, 84 mice with clean hair removal and no skin damage were selected and randomly divided into the blank group, ointment group, minoxidil group, CE-NE (25 mg/kg, 50 mg/kg and 100 mg/kg), with 12 animals in each group, established the pathological alopecia model. Apply the liquid twice a day until the hair grows out of the hair removal area on the back.

### 4.7. Evaluation of Hair Growth Cycle

During the application period, no difference or abnormalities in the average body weight of all mice was observed. The skin and coat growth of mice were observed every day, and the time when the skin color of each mice’s hair removal area changed from pink to gray, and from gray to hairy was recorded. The hair regrowth activities of cedrol were evaluated by observing and photographing the back skin of mice. The assessment of changes in skin color change during different growth cycles was quantified using grayscale values (select the day 12 to calculate). The 12 days grayscale values and divided by the control skin grayscale values was measured as the each experimental group using the Image J software.

### 4.8. Determination of the Hair Length and Weight

The skin and hair growth of mice were observed and photographed every day. Researchers chose the 5–10 hairs were randomly extracted from the same part of the back of each mouse and measured under the microscope. The length of the hair was recorded at the furthest distance between the two ends of the hair to calculate the average length. The data were analyzed by GraphPad Prism software. (Allianz Business Consulting (Beijing) Co., Ltd, Version 5, Beijing, China)

Mice were sacrificed by cervical dislocation after 21 days. Each group of mice selected the same location of the back skin area 0.4 cm^2^ areas, and calculated the gross weight.

### 4.9. Pathological Section Observation

After being treated with drugs every day for 21 days, the back skin of different groups of mice in the same position was removed with surgical scissors. The skin pieces were fixed in 10% formaldehyde solution, washed with water, two pieces of tissue, each 1.5 cm long, were taken along the longitudinal section of the hair follicle, and routine tissue dehydration, paraffin embedding and HE staining (hematoxylin-eosin staining). The length of hair follicles was measured by an optical microscope Nikon (NIKON INSTRUMENTS (SHANGHAI) CO., LTD, Shanghai, China). The skin histological changes of mice hair follicles were observed under light microscope and the hair follicle length was measured.

### 4.10. Bioavailability Studies

#### 4.10.1. Chromatography and Mass Spectrometry Conditions

Chromatographic separation was performed by using a 7890 B gas chromatographic system combined with a 7000 triple quadrupole mass spectrometer (GC-MS, Agilent, Palo Alto, USA). Chromatographic separation was achieved on a HP-5MS column (30 m × 0.25 mm × 0.25 μm) purchased from America.

Cedrol was analyzed by GC-MS using nitrogen as the carrier gas with a flow of 1.0 mL min^−1^. The initial column temperature was held at 130 °C for 2 min and then increased at a rate of 15 °C min^−1^ to 210 °C for 1 min. The mass spectrometer was operated in positive electron impact ionization mode (70 eV). The MS ion source temperatures were set at 230 °C. The quantification was performed using selected ion detection (SIM): *m*/*z* 95,150 and 151 for cedrol, *m*/*z* 109 and 74.02 for IS [30].

#### 4.10.2. Pharmacokinetic Studies

The tests were performed using KM mice (*n* = 5). The day before the experiment, an area of 2 cm × 2 cm was removed from the dorsal skin of the mice. The animals were divided into two groups: A and B, cedrol ointment and the optimized CE-NE (50 mg kg) were applied on the dorsal prepared region of the animals. The selected mice were fixed, without anesthesia. Mice were sacrificed at different time points: 0.25 h, 0.5 h, 1 h, 2 h, 4 h, 6 h, 8 h, 10 h, 12 h, and 24 h. The remaining formulation was removed by a cotton swab impregnated with physiological saline. The stratum corneum, subcutaneous fat and connective tissue of the skin samples were removed with adhesive tape and surgical shears. Mice skin was collected and the bio-samples were stored at −20 °C until analysis.

Each sample was homogenized with 1.0 mL of saline with IS, then 1.0 mL of *n*-hexane was added and vortex mixed for 3 min. The samples were ultrasonicated for 5 min and centrifuged at 14,000 rpm for 5 min. 1.0 μL of supernatant was injected for GC-MS analysis. The measured values were brought into the regression equation to calculate the drug concentration at each time point. The pharmacokinetic parameters were calculated by DAS 2.0 software and the statistical moment method was used to calculate the pharmacokinetic parameters after single administration.

### 4.11. Statistical Analysis

Results were shown as mean standard deviation (S.D). Data analyses were analyzed by Student’s *t*-test (GraphPad Prism for Windows, Allianz Business Consulting (Beijing) Co., Ltd, Version 5, Beijing, China) for the significance of the calculation results (* *p* < 0.05, ** *p* < 0.01, *** *p* < 0.001).

## 5. Conclusions

In summary, the results showed that CE-NE significantly promoted hair growth. Compared with ointment and 2% minoxidil groups, 50 mg/mL CE-NE group had stronger activity in promoting hair growth. In addition, the area of the concentration-time curve (AUC) (0–*t*) of the CE-NE (330.30 ± 23.07 μg/mL·h) was 4-fold higher than that of the ointment (82.56 ± 6.52 μg/mL·h). From this date, we suggest that CE-NE represents the most promising effects as a hair growth promoter.

## Figures and Tables

**Table 1:** Cumulative release curve fitting results of CE-NE in vitro (pH 4.0).

Model	Equations	R^2^
Zero-order kinetic equation	y = 0.00914 + 0.06135x	0.27012
First-order kinetic equation	y = 35.40868 ∗ (1 − e^(−0.7177x)^)	0.98543
Higuchi	y = 4.53386 ∗ x^0.5^ − 15.75977	0.56208
Korsmeyer–Peppas	y = 19.95596 ∗ x^0.20777^	0.77740

**Table 2:** Cumulative Release Curve Fitting Results of CE-NE In Vitro (pH 7.8).

Model	Equations	R^2^
Zero-order kinetic equation	y = 7.59352 + 0.43895x	0.53682
First-order kinetic equation	y = 22.59151 ∗ (1 − e^(−0.15833x)^)	0.99736
Higuchi	y = 3.85499 ∗ x^0.5^ − 1.96123	0.82278
Korsmeyer–Peppas	y = 6.39378 ∗ x^0.37049^	0.87818

**Table 3:** Effect of cedrol on qualitative hair growth.

Treatment	Hair Growth (Days)		
	Skin from Pink to Grey Time	Initiation Time	Short-Hair Complete Time
Control	8.3 ± 0.8	11.7 ± 0.5	14.7 ± 0.5
CE-NE (25 mg/kg)	6.7 ± 0.5 **	10.7 ± 0.8 **	12.8 ± 0.4 **
CE-NE (50 mg/kg)	6.2 ± 0.4 ***	9.3 ± 0.5 ***	11.8 ± 0.4 ***
CE-NE (100 mg/kg)	10.5 ± 1.1 ***	16.5 ± 0.6 **	20.0 ± 0.9 ***
Ointment	6.75 ± 0.6 **	10.6 ± 0.5 ***	12.5 ± 0.5 ***
2% Minoxidil	7.5 ± 0.8 **	10.0 ± 0.6 ***	12.3 ± 0.5 ***

Hair-growth initiation and completion time were recorded for each group of mice. The results were shown as the mean values ± S.D. ** *p* < 0.01, *** *p* < 0.001, when compared to respective control values by Student’s *t*-test (*n* = 10).

**Table 4:** Hair Length of Mice at 13, 18 and 21 Days.

Gender in Mice	Treatment	Hair Length (mm)		
		13 Day	18 Day	21 Day
Female	Control	3.5 ± 0.8	6.5 ± 0.8	9.2 ± 0.7
	CE-NE (25 mg/kg)	5.3 ± 0.4 **	8.3 ± 0.4 **	10.6 ± 0.3 **
	CE-NE (50 mg/kg)	5.6 ± 0.2 ***	8.6 ± 0.2 ***	11.3 ± 0.7 ***
	Ointment	4.8 ± 0.6 **	8.3 ± 0.4 **	10.5 ± 0.7 *
	2% Minoxidil	5.1 ± 0.2 *	8.3 ± 0.3 *	10.6 ± 0.7 **
	CE-NE (100 mg/kg)			3.8 ± 0.3

Length of hair at different time intervals after beginning the treatment of CE-NE, 2% minoxidil and ointment. The results were shown as the mean ± S.D. * *p* < 0.05, ** *p* < 0.01, *** *p* < 0.001, when compared to respective control values by Student’s *t*-test (*n* = 10).

**Table 5:** Effect of cedrol on hair weight.

Gender in Mice	Treatment	Hair Weight (mg)
		21 Day
Female	Control	0.22 ± 0.06
	CE-NE (25 mg/kg)	0.41 ± 0.08 **
	CE-NE (50 mg/kg)	0.54 ± 0.04 ***
	CE-NE (100 mg/kg)	0.20 ± 0.31
	Ointment	0.44 ± 0.04 **
	2% Minoxidil	0.4 ± 0.04 **

Weight of hair dorsal skin at different groups after 21 days. The results were shown as the mean ± S.D. ** *p* < 0.01, *** *p* < 0.001, when compared to respective control values by Student’s *t*-test (*n* = 10).

**Table 6:** Effect of cedrol on length of hair follicle.

Treatment	Length of Hair Follicle (μm)
	21 Day
Control	55.22 ± 11.34
CE-NE (25 mg/kg)	209.99 ± 30.68
CE-NE (50 mg/kg)	236.18 ± 31.08
CE-NE (100 mg/kg)	85.85 ± 15.87
Ointment	147.24 ± 27.45
2% Minoxidil	198.05 ± 22.11

Length of hair follicles at 21 days after beginning the treatment of CE-NE, 2% minoxidil and ointment. The results were shown as the mean ± S.D., when compared to respective control values by Student’s *t*-test (*n* = 10).

**Table 7:** Pharmacokinetic parameters of CE-NE and ointment after topical administration at a dose of 50 mg/kg in KunMing mice (*n* = 5).

Parameters	Ointment	CE-NE
AUC0-24 (μg/mL·h)	82.56 ± 6.52	330.296 ± 23.07 **
AUC0-∞ (μg/mL·h)	177.40 ± 93.27	355.506 ± 28.74 **
MRT (0–*t*) (h)	6.717 ± 0.17	9.63 ± 1.041
Cmax (μg/mL)	11.30 ± 1.70	46.83 ± 3.15 **
Tmax (h)	1	1
CL (L/h/kg)	353.35 ± 178.55	141.44 ± 12.30

The results were shown as the mean ± S.D. ** *p* < 0.01, when Compared to the ointment group by Student’s *t*-test.

## Data Availability

Data available in a publicly accessible repository.

## Sample Availability

Samples of the compounds are available from the authors.
